# Screening of immigrants in the UK for imported latent tuberculosis: a multicentre cohort study and cost-effectiveness analysis

**DOI:** 10.1016/S1473-3099(11)70069-X

**Published:** 2011-06

**Authors:** Manish Pareek, John P Watson, L Peter Ormerod, Onn Min Kon, Gerrit Woltmann, Peter J White, Ibrahim Abubakar, Ajit Lalvani

**Affiliations:** a Department of Infectious Disease Epidemiology, Imperial College London, London, UK; b Tuberculosis Research Unit, National Heart and Lung Institute, Imperial College London, London, UK; c Department of Respiratory Medicine, Leeds Teaching Hospitals NHS Trust, Leeds, UK; d Chest Clinic, Royal Blackburn Hospital, Blackburn, UK; e Tuberculosis Service, Chest and Allergy Clinic, St Mary's Hospital, Imperial College Healthcare NHS Trust, London, UK; f Institute for Lung Health, University Hospitals Leicester NHS Trust, Leicester, UK; g Modelling and Economics Unit, Centre for Infections, Health Protection Agency, London, UK; h Tuberculosis Section, Centre for Infections, Health Protection Agency, London, UK

## Abstract

**Background:**

Continuing rises in tuberculosis notifications in the UK are attributable to cases in foreign-born immigrants. National guidance for immigrant screening is hampered by a lack of data about the prevalence of, and risk factors for, latent tuberculosis infection in immigrants. We aimed to determine the prevalence of latent infection in immigrants to the UK to define which groups should be screened and to quantify cost-effectiveness.

**Methods:**

In our multicentre cohort study and cost-effectiveness analysis we analysed demographic and test results from three centres in the UK (from 2008 to 2010) that used interferon-γ release-assay (IGRA) to screen immigrants aged 35 years or younger for latent tuberculosis infection. We assessed factors associated with latent infection by use of logistic regression and calculated the yields and cost-effectiveness of screening at different levels of tuberculosis incidence in immigrants' countries of origin with a decision analysis model.

**Findings:**

Results for IGRA-based screening were positive in 245 of 1229 immigrants (20%), negative in 982 (80%), and indeterminate in two (0·2%). Positive results were independently associated with increases in tuberculosis incidence in immigrants' countries of origin (p=0·0006), male sex (p=0·046), and age (p<0·0001). National policy thus far would fail to detect 71% of individuals with latent infection. The two most cost-effective strategies were to screen individuals from countries with a tuberculosis incidence of more than 250 cases per 100 000 (incremental cost-effectiveness ratio [ICER] was £17 956 [£1=US$1·60] per prevented case of tuberculosis) and at more than 150 cases per 100 000 (including immigrants from the Indian subcontinent), which identified 92% of infected immigrants and prevented an additional 29 cases at an ICER of £20 819 per additional case averted.

**Interpretation:**

Screening for latent infection can be implemented cost-effectively at a level of incidence that identifies most immigrants with latent tuberculosis, thereby preventing substantial numbers of future cases of active tuberculosis.

**Funding:**

Medical Research Council and Wellcome Trust.

## Introduction

Although tuberculosis prevails in mainly high-burden developing countries, cases in immigrants in many low-incidence countries are increasing substantially.^1^ This changing pattern of disease is clear in the UK where, between 1998 and 2009, tuberculosis notifications have risen by 46%, from 6167 cases to 9040, with much of this rise fuelled by the 98% increase in cases from overseas.^2^, ^3^ These individuals account for nearly three-quarters of all tuberculosis notifications in the UK with an incidence that is 20 times higher than in UK-born individuals (89 cases per 100 000 people per year *vs* 4 per 100 000).^3^

The evolving epidemiology in high-income countries is driven mostly by migration of individuals from countries with a high burden of disease, such as sub-Saharan Africa and the Indian subcontinent,^4^, ^5^ and by the reactivation of latent tuberculosis infection that was acquired before migration.^6^ These factors result in a high incidence of tuberculosis in immigrants in the first 2–5 years after migration (with about 50% of foreign-born cases presenting in the first 5 years after migration), which then decreases over time.^7^, ^8^

Changes in incidence have renewed interest in tuberculosis screening of immigrants.^9^ Data in several high-income countries suggest that screening for latent infection is highly variable—both in which immigrants are screened, and how they are screened.^10^ UK national policy specifies port-of-entry identification and screening with chest radiographs for immigrants from countries with a tuberculosis incidence of more than 40 cases per 100 000 population per year who intend to stay in the UK for more than 6 months. The aim of this initial screening is to detect active pulmonary tuberculosis,^11^ and results determine the subsequent actions taken by the individual's local tuberculosis services.

Actions should be undertaken in line with national guidelines for tuberculosis control.^12^ For most immigrants with normal chest radiographs, since 2006, the National Institute for Health and Clinical Excellence (NICE) recommends that local tuberculosis services should screen specific subgroups of new entrants for latent infection, including children aged less than 16 years from countries with a tuberculosis incidence or more than 40 per 100 000 per year, and 16–35-year-olds from either sub-Saharan countries or from those with a disease incidence of more than 500 per 100 000 per year. Individuals older than 35 years are not screened because the risks of chemoprophylaxis outweigh the potential benefits.^12^

The rationale supporting this screening approach remains unclear, especially because data are scarce for the prevalence of latent infection in new immigrants as measured by interferon-γ release assays (IGRAs).^13^ Furthermore, although NICE's recommendation for the two-step method of screening (ie, tuberculin skin-test plus confirmatory IGRA) has been adopted in most European countries, the USA,^14^ and many centres in the UK increasingly use one-step IGRA testing to screen for latent infection,^13^, ^15^ probably because of this test's high specificity.^16^, ^17^, ^18^ Additional reasons for the use of the one-step test include evidence that IGRAs might be able to predict the development of active tuberculosis from latent infection,^19^, ^20^, ^21^, ^22^, ^23^, ^24^, ^25^, ^26^, ^27^, ^28^ and uncertainty about the optimum cutoff for a positive skin test in the context of previous BCG vaccination.^29^ We did this multicentre cohort study to compute yields from, and cost-effectiveness of, screening for latent infection at different thresholds in relation to incidence of tuberculosis in immigrants' countries of origin.

## Methods

### Study design and participants

We did this prospective multicentre study and cost-effectiveness analysis of immigrant screening in three centres in the UK: Westminster, London; Leeds, Yorkshire; and Blackburn, Lancashire. Together these centres serve 1·6 million people^30^ of whom 6·5% (IQR 4·3%–9·9%) are foreign born.^31^ Between 2007 and 2009, the average 3-year notifications in these centres ranged from 54 to 126, and incidence varied from 16 to 33 cases per 100 000 population per year.^32^

Participants were foreign-born new entrants (arrival within the past 5 years) who were aged 35 years or younger and who were referred for and underwent tuberculosis screening between Jan 1, 2008, and July 31, 2010. Referrals to these centres were made either through port-of-entry screening systems, health-protection units, or after registration with primary-care services. Ethical approval was not needed because the study used fully anonymised observational data that were obtained as part of an assessment of routine clinical service.

### Screening and management

We first screened immigrants who attended the centres with a symptom questionnaire followed by one-step IGRA (QuantiFERON-TB Gold In-Tube. Carnegie, Cellestis, Australia), a whole blood ELISA, containing ESAT-6 (early secretory antigenic target-6), CFP-10 (culture filtrate protein-10), and TB7.7 (Rv2654), which was done in accordance with the manufacturer's instructions. Results were positive, negative, or indeterminate, dependent on the manufacturer's criteria. A meta-analysis^33^ of the effectiveness of the QuantiFERON-TB Gold In-Tube suggests that sensitivity is 84% and specificity is 99%. Immigrants who were symptomatic or who had a positive IGRA result were referred for chest radiography and further clinical assessment to discount active tuberculosis.^12^

We defined latent tuberculosis infection as immigrants with a positive IGRA and normal chest radiography in the absence of any clinical features that would suggest active disease.^34^ Immigrants who were diagnosed with latent infection were offered chemoprophylaxis with either 3 months of rifampicin and isoniazid, or 6 months of isoniazid, in accordance with UK guidelines,^12^ dependent on clinician and patient preference.

### Statistical analysis

We obtained data for demographics (age categorised as <16 years, 16–25 years, or 26–35 years, and sex), BCG vaccination status (ascertained through documentary evidence, reliable history of vaccination, or a characteristic scar^35^), and country of origin. From reported country of origin, we further classified data into region of origin (Europe and the Americas, Middle East and north Africa, other Asia, Indian subcontinent, or sub-Saharan Africa) and we took tuberculosis incidence in the country (categorised as 0 cases per 100 000/year–50 cases per 100 000/year; 51/100 00–150/100 000; 151/100 000–250/100 000; 251/100 000–350/100 000, and ≥350/100 000) from WHO's 2009 global report on tuberculosis.^36^

Continuous data were summarised with median and IQR, and were compared with the non-parametric Mann-Whitney U-test. Categorical responses were expressed as a simple descriptive percentage with 95% CIs, and comparisons were made with Pearson χ^2^ or Fisher's exact test as appropriate. We calculated yield of latent infection as the proportion of individuals who were IGRA positive; indeterminate results were included in the denominator when calculating IGRA-positivity. We assessed univariate associations of the presence of latent infection with age, sex, region of origin, tuberculosis incidence in country of origin, and BCG status using logistic regression, and reported as crude odds ratios (OR) and 95% CIs. We then calculated adjusted ORs by mutually adjusting in a multivariate logistic regression for age, sex, and tuberculosis incidence in country of origin (to account for potential confounders) with the same categories outlined above. We did not include BCG status in the multivariate model because of the high proportion of missing values.

To assess the different thresholds of incidence screening we calculated, at every incidence level cutoff, the absolute number of immigrants needing to be screened, the yield for latent tuberculosis infection, and the proportion of individuals with latent infection who would not be detected at particular thresholds of screening. Because screening of children is a priority in tuberculosis control, and because the number of child immigrants younger than 16 years is small, we also considered screening all children irrespective of tuberculosis incidence in their country of origin.

Analyses used STATA version 9.2. All tests were two-tailed and p values less than 0·05 were regarded as significant.

### Economic analysis

Economic analysis was done from a UK National Health Service perspective to consider two main questions related to use of a one-step IGRA strategy over 20 years. What are the costs of screening at different incidence thresholds? And is screening at specific thresholds cost effective and, if so, which threshold if any is the most cost effective? We developed a decision tree (webappendix pp 12–15) to simulate the clinical (number of cases of active tuberculosis), and economic outcomes of screening a hypothetical cohort of 10 000 new immigrants aged 35 years and younger for latent infection over a 20-year timeline.

We considered screening using QuantiFERON-TB Gold In-Tube alone and varying the incidence threshold in the country of origin at which individuals became eligible for screening. At each threshold cutoff, we assessed the number of immigrants who would be eligible for screening, the number who would be IGRA-positive, and the number of IGRA-positives that would be undetected compared with screening of the whole cohort. The decision tree was constructed and analysed with Microsoft Excel 2007 and TreeAge Pro 2011 (Tree Age Software, Williamstown, MA, USA). Panel 1 shows the model assumptions. For descriptions and discussion of the decision model, sources for the associated costs (in pounds sterling) and input probabilities and parameters, how cost-effectiveness was measured, and ranges for sensitivity analysis see webappendix (pp 2–9).

Panel 1 **Model assumptions of the health economic model**

• Immigrants are screened for latent tuberculosis infection at the start of the 20-year time line
• All IGRA results are determinate and no repeat testing is needed
• At the time of screening the immigrants there are no prevalent cases of active tuberculosis in the cohort
• There are no HIV-coinfected individuals in the cohort
• All active cases are caused by a tuberculosis strain that is fully drug sensitive
• In individuals with latent infection who are treated with chemoprophylaxis, a 3-month course of rifampicin and isoniazid has the same effectiveness as 6 months of isoniazid
• Individuals who start chemoprophylaxis and subsequently develop drug-induced liver injury that does not resolve are assumed to complete only 4 weeks of therapy, which affords no reduction in the risk of progressing to active infection
• An individual with latent tuberculosis who has completed successful chemoprophylaxis is assumed to have cleared the infection with *Mycobacterium tuberculosis* and will not experience any further outcomes in the time course of the model
• An individual who does not have latent infection on arrival in the UK does not become infected during the period of the model
• Data for the test performance of the IGRA were based on the most recent meta-analysis obtained from meta-analyses in which sensitivity was calculated with culture-confirmed active tuberculosis as the reference standard; specificity was calculated from BCG-vaccinated individuals at low risk of infection
• All individuals who are diagnosed with active tuberculosis are assumed to accept treatment for active infection and to complete the 6-month course of drugs

### Role of the funding source

The funding sources played no part in the study design, data analysis, writing of the manuscript or decision to submit for publication. None of the investigators were paid to write this article by a pharmaceutical company or other agency. The corresponding author had full access to the data and had final responsibility for the decision to submit for publication.

## Results

**Figure 1:**
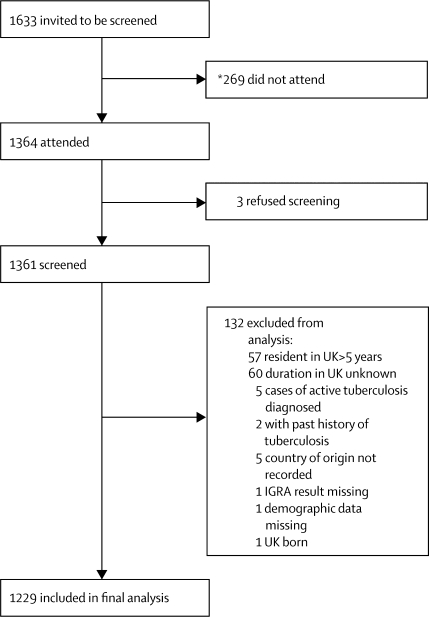
Study flow diagram *Data for non-attendees available for only two of the three centres in the study.

Recruitment into the study is outlined in figure 1. Table 1 shows the demographics of the screened population (n=1229). 1193 (97%) of screened immigrants were mostly young adults (aged 16–35 years) and attendees were less likely to be male than female (odds ratio [OR] 0·6, 95% CI 0·5–0·9). Data for previous BCG vaccination were available for only 657 participants, of whom about 80% had been vaccinated. Screened immigrants most commonly originated from the Indian subcontinent and sub-Saharan Africa; Pakistan and India were the most common countries of origin (32% and 26%, respectively). Overall, the screened immigrants were broadly representative of the foreign-born population in the UK; however, our study population contained slightly more immigrants from the Indian subcontinent, and slightly fewer from sub-Saharan Africa than the national average.^31^

**Table 1:** Demographics of cohort and risk factors associated with IGRA positivity in immigrants

	**Number in total cohort (n=1229)**	**Number of IGRA-positive individuals/total number tested (n=245)**	**Unadjusted OR (95% CI)**	**p value**	**Adjusted OR (95% CI)**	**p value**
**Age (years)**						
<16*	36 (3%)	7/36 (19%)	1	0·0051†	1‡	<0·0001§
16–25	589 (48%)	86/589 (15%)	0·7 (0·3–1·7)	..	0·9 (0·4–2·1)	..
26–35	604 (49%)	152/604 (25%)	1·4 (0·6–3·2)	..	1·7 (0·7–4·1)	..
**Sex**						
Female	629 (51%)	109/629(17%)	1	0·02	1¶	0·046
Male	600 (49%)	136/600 (23%)	1·4 (1·1–1·9)	..	1·3 (1·0–1 8)	..
**Origin**‖						
Europe, Americas	50 (4%)	2/50 (4%)	1	0·0011	..	..
Middle East, North Africa	26 (2%)	1/26 (4%)	1·0 (0·1–11·1)	..	..	..
Other Asia	162 (13%)	29/162 (18%)	5·2 (1·2–22·8)	..	..	..
Indian subcontinent	740 (60%)	144/740 (20%)	5·8 (1·4–24·1)	..	..	..
Sub-Saharan Africa	251 (20%)	69/251 (28%)	9·1 (2·2–38·5)	..	..	..
**Incidence of tuberculosis in country of origin (cases per 100 000 population per year)**‖						
0–50	32 (3%)	1/32 (3%)	1	<0·0001†	1**	0·0006
51–150	150 (12%)	19/150 (13%)	4·5 (0·60–34·9)	..	4·5 (0·60–35·3)	..
151–250	835 (68%)	164/835 (20%)	7·6 (1·0–55·9)	..	7·9 (1·1–58·3)	..
251–350	139 (11%)	41/139 (30%)	13·0 (1·7–98·2)	..	13·3 (1.8–101·5)	..
>350	73 (6%)	20/73 (27%)	11·7 (1·5–91·5)	..	13·1 (1·7–102·7)	..
**BCG vaccinated?**						
No	113 (17%)	16/113 (14%)	1	0·17	..	..
Yes	544 (83%)	107/544 (20%)	1·5 (0·8–2·6)	..	..	..

IGRA=interferon-γ release assay. OR=odds ratio.

* Of the 36 individuals aged <16 years, one (2·8%) was aged ≤4 years, one (2·8%) was 5–9 years, and 34 (94·4%) were 10–15 years.

† χ^2^ p for trend.

‡ Mutually adjusted for sex and incidence of tuberculosis in country of origin.

§ p value denotes overall effect of age in the model.

¶ Mutually adjusted for age and tuberculosis incidence in country of origin.

‖ Region of origin and tuberculosis incidence in country of origin were strongly correlated; therefore, in the multivariate analysis, region of origin was left out.

** Mutually adjusted for age and sex.

**Figure 2:**
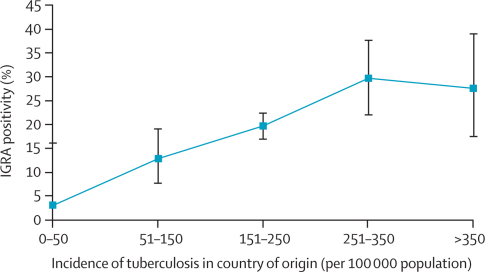
Proportion of immigrants aged 35 years or younger who tested IGRA positive according to tuberculosis incidence in their country of origin IGRA=interferon-γ release assays. Bars=95% CIs.

IGRA results were available for all participants. Overall, 245 individuals tested positive (20%, 95% CI 18–22%), 982 were negative (80%, 77–82%), and two had indeterminate results (<1%, 0–1%). Participants attending the Westminster centre had a significantly lower proportion of IGRA-positive results than did those attending the Leeds and Blackburn centres (p=0·02). The proportions of positive immigrants aged less than 16 years, 16–25 years, and 26–35 years were 19%, 15%, and 25%, respectively. In multivariate analysis, male sex, increasing age, and tuberculosis incidence in country of origin were associated with positive IGRA (table 1, figure 2).

Table 2 outlines the outcomes of immigrant screening for latent infection stratified by age and incidence in the immigrants' countries of origin. In all age groups, as the incidence threshold at which screening is instigated increases, fewer immigrants within the cohort are eligible to be screened and, consequently, the number of identified latent cases also decreases.

**Table 2:** Yields for latent tuberculosis infection (defined as positive QuantiFERON assay) for different age groups and at different screening thresholds of incidence in country of origin

	**Number tested**	**Number positive**	Yield at incidence level*	**Proportion of all latent infection identified if threshold set at this level**
**Aged <16 years**†				
Screen ≥500 and sub-Saharan Africa	16	4	25·0%	57·1%
Screen ≥500	6	2	33·3%	28·6%
Screen ≥450	6	2	33·3%	28·6%
Screen ≥400	6	2	33·3%	28·6%
Screen ≥350	7	2	28·6%	28·6%
Screen ≥300	12	2	16·7%	28·6%
Screen ≥250	15	3	20·0%	42·9%
Screen ≥200	23	4	17·4%	57·1%
Screen ≥150	34	6	17·7%	85·7%
Screen ≥100	34	6	17·7%	85·7%
Screen ≥40‡	36	7	19·4%	100%
Screen all	36	7	19·4%	100%
**16–35 years**†				
Screen ≥500 and sub-Saharan Africa‡	235	65	27·7%	27·3%
Screen ≥500	46	12	26·1%	5·0%
Screen ≥450	54	13	24·1%	5·5%
Screen ≥400	55	13	23·6%	5·5%
Screen ≥350	66	18	27·3%	7·6%
Screen ≥300	135	38	28·2%	15·9%
Screen ≥250	197	58	29·4%	24·4%
Screen ≥200	668	127	19·0%	53·4%
Screen ≥150	1013	219	21·6%	92·0%
Screen ≥100	1068	222	20·8%	93·3%
Screen ≥40	1180	238	20·2%	100%
Screen all	1193	238	20·0%	100%

* Proportion of those tested giving a positive result.

† Per 100 000 population per year.

‡ Present NICE guidance.

Application of NICE guidance to our cohort would result in 271 individuals out of 1229 (22%) being eligible for screening, of whom 72 (27%) were IGRA positive, representing 29% of all cases of latent infection. Decreasing the screening threshold for adults to 150 cases per 100 000 (with the threshold for individuals aged <16 years unchanged) increases the number of immigrants who are eligible for screening to 1049 (85%) of 1229 (p<0·0001), with a similar proportionate yield of 226 out of 1049 (22%) and significantly more latent cases identified (92%, p<0·0001) than with NICE guidance.

Table 3 shows the results of the health-economic analysis, including the predicted number of tuberculosis cases and associated costs for each protocol in a cohort of 10 000 immigrants over 20 years. Although strategies that used screening with IGRA were more expensive than no screening, they also resulted in fewer cases of active tuberculosis in the 20-years. Costs increased as the threshold of incidence in country of origin at which immigrants were eligible to be screened fell (table 3). Screening of all immigrants aged 35 years and under from any countries irrespective of tuberculosis incidence would cost more than £1·5 million and prevent 44·5 cases of tuberculosis, whereas application of NICE guidance would cost about £850 000 and prevent 13·2 cases of active disease. Although no immigrant screening for latent tuberculosis infection was the least expensive option (£600 000), it resulted in the most cases of active tuberculosis.

**Table 3:** Projected cases of active tuberculosis and associated costs with screening immigrants at different thresholds of tuberculosis incidence

	**Cases of active tuberculosis over 20 years**	**Costs over 20 years (2010 £)**	Incremental cases of active tuberculosis prevented over 20 years*	**Incremental costs over 20 years**†**(£)**	**ICER (£ per tuberculosis case prevented)**
**No screening <16 year olds**					
Screen 0 16–35 year olds	95·4	608 370·0	Baseline	Baseline	Baseline
**Screen <16 year olds, 40**‡					
Screen 16–35 year olds, 500	91·9	678 586·5	Extended dominance	Extended dominance	Extended dominance
Screen 16–35 year olds, 400	91·8	683 710·0	Strict dominance	Strict dominance	Strict dominance§
Screen 16–35 year olds, 450	91·7	683 267·9	Extended dominance	Extended dominance	Extended dominance¶
Screen 16–35 year olds, 350	90·8	697 208·7	Extended dominance	Extended dominance	Extended dominance
Screen 16–35 year olds, 300	87·1	761 431·6	Extended dominance	Extended dominance	Extended dominance
Screen 16–35 year olds, 250	83·4	823 312·8	12·0	214 942·8	17 956·0
Screen 16–35 year olds + sub-Saharan Africa, 500‖	82·2	850 103·1	Extended dominance	Extended dominance	Extended dominance
Screen 16–35 year olds, 200	71·1	1 121 093·2	Extended dominance	Extended dominance	Extended dominance
Screen 16–35 year olds, 150**	54·2	1 431 928·5	29·2	608 615·7	20 818·8
Screen 16–35 year olds, 100	53·7	1 456 820·1	Extended dominance	Extended dominance	Extended dominance
Screen 16–35 year olds, 40	50·9	1 527 478·5	3·2	95 550·1	29 403·1
**Screen all <16y**					
Screen all 16–35 year olds	50·9	1 532 256·6	0·0	4778·0	101 938·3

Arranged in order of increasing effectiveness—ie, fewer cases of active tuberculosis for a hypothetical cohort of 10 000 immigrants over 20 years. When different strategies are ranked from least effective to most effective (ie, number of cases of active tuberculosis that are predicted to occur), the incremental cost-effectiveness ratios (ICER) of most screening options, including present National Institute for Health and Clinical Excellence (NICE) guidance, are excluded through extended dominance.

* Incremental number of cases are calculated as the difference (ie, number of cases prevented) from the previous non-dominated option.

† Incremental costs are calculated as the difference (ie, extra £) from the previous non-dominated option.

‡ Incidence per 100 000 per year.

§ Strict dominance—by which a particular screening threshold is both less effective and more expensive than the next most effective screening threshold.

¶ Extended dominance—by which the ICER for a particular screening threshold is higher than for the next most effective strategy (screening threshold), therefore, the higher ICER is removed from the cost-effectiveness analysis.

‖ Represents the situation occurring if screening is done by UK national (NICE) guidance.

** The situation occurring if screening included immigrants from the Indian subcontinent.

After exclusion of dominated strategies (table 3), four cost-effective strategies remained. In decreasing order of cost-effectiveness, these strategies were (in addition to screening immigrants younger than 16 years from countries ≥40/100 000) to screen 16–35 year olds from countries with incidences of 250 per 100 000 and higher, 150 per 100 000 and higher, and more than 40 per 100 000. The fourth strategy was to screen all individuals aged 35 years and younger from all countries irrespective of tuberculosis incidence. The associated ICERs were £17 956·0, £20 818·8, £29 403·1, and £101 938·3, respectively, per active case averted. Therefore, for ICERs, the most cost-effective strategies would be to start screening at 40 cases per 100 000 for individuals aged less than 16 years, and 250 per 100 000 for 16–35 year olds. However, the ICER for the next most cost-effective strategy (screening individuals aged less than 16 years at 40 per 100 000 and 16–35-year-olds at 150 per 100 000) was only just under £3000 higher than the most cost-effective strategy. Strategies to further reduce the threshold to include screening all immigrants aged 35 years and younger from countries with incidences of 40 cases per 100 000 and higher, or indeed all immigrants, were both non-dominated options; however, the associated ICERs were very high.

Numbers needed to screen and numbers needed to treat (NNT) ranged from 165·5 to 231·9 and 42·0 to 42·7 respectively. Screening 16–35-year-olds at 250 per 100 000 had the lowest NNT (42·0), whereas screening at a threshold higher than this value generally resulted in a higher NNT.

Table 4 and webappendix (p 10–11) show results of the univariate sensitivity analysis. Changing several of the variables affected estimates for the ICERs of each of the strategies but did not significantly affect the rank order of the most cost-effective strategies. The most important variables were the rate at which new-entrants progress to active tuberculosis and the prevalence of latent tuberculosis in the screened cohort. Increased values for both variables increased cost-effectiveness (ie, lower ICERs). Cost-effectiveness was significantly more affected by diagnostic specificity than by sensitivity. Reductions in specificity increased ICER estimates (ie, reduced cost-effectiveness) because more false-positive, uninfected individuals would be treated unnecessarily. Reductions in screening costs for latent infection, or assessment of those who screened positive, significantly reduced ICER values (ie, increased cost-effectiveness).

**Table 4:** Univariate sensitivity analysis of the probabilities and proportions that were used as input variables in the decision model

	**Point estimate**	**Range explored**	<16 years >40* 16–35 years >250	**<16 years >40** **16–35 years >150**	**<16 years >40** **16–35 years >40**	**Screen all <16 years** **Screen all 16–35 years**
Prevalence of latent tuberculosis infection	0·22	0·1 0·4	28 853·7 14 527·9	36 319·9 16 081·0	61 481·5 20 650·3	136 739·3 73 864·9
Sensitivity of IGRA	0·84	0·78 0·90	17 932·7 17 973·1	20 788·3 20 841·2	29 442·5 29 374·3	86 066·0 117 507·2
Specificity of IGRA	0·99	0·88 1·00	29 372·8 17 365·2	50 789·5 19 751·4	SD 26 108·0	SD 272 290·5
Proportion progressing to active tuberculosis (over 20 years)	0·05	0·025 0·15	41 823·6 2049·3	47 494·4 3 040·8	64 498·4 6 013·6	208 178·6 31 133·8
Number of contacts	6·5	3·25 10	19 522·5 16 269·0	22 385·3 19 131·8	30 969·6 27 716·1	103 504·8 100 251·3
Efficacy of complete chemoprophylaxis (RR %)	0·65	0·5 0·8	24 772·0 13 587·3	28 418·9 15 943·5	39 717·1 22 860·8	161 114·4 85 522·9
Effectiveness of partial chemoprophylaxis (RR %)	0·21	0·1 0·3	18 654·1 17 413·2	21 597·6 20 213·3	30 453·4 28 587·3	99 352·9 104 149·4
Proportion starting chemoprophylaxis	0·95	0·3 1·0	60 149·2 16 985·3	68 786·9 19 710·9	SD 27 841·5	98 102·9 107 085·1
Proportion of individuals completing chemoprophylaxis	0·85	0·3 1·0	32 756·6 15 836·0	37 561·9 18 417·1	53 089·1 26 072·5	554 774·1 91 930·3
Number of secondary cases of active tuberculosis per index case	0·2	0·1 0·3	20 162·9 16 088·1	23 285·6 18 731·1	32 648·9 26 655·9	111 673·0 93 539·9
Number of secondary cases of latent tuberculosis cases per index case	0·18	0·09 0·27	17 983·4 17 928·6	20 848·5 20 789·3	29 439·3 29 366·9	102 030·7 101 846·0
Proportion of active cases admitted as inpatient	0·53	0·265 0·795	19 019·4 16 892·6	21 882·3 19 755·4	30 466·5 28 339·6	103 001·7 100 874·9
Proportion of immigrants receiving chemoprophylaxis who developed drug-induced liver injury	0·002	0·001 0·003	17 944·4 17 967·6	20 808·4 20 829·2	29 396·1 29 410·0	101 895·2 101 981·5

Only non-dominated options are presented. The figures presented are the incremental cost-effectiveness ratios (ICERs). Increasing ICER indicates decreasing cost-effectiveness.

* Incidence per 100 000 per year. IGRA=interferon-γ release assay. SD=strict dominance. RR=risk reduction.

## Discussion

Our assessment of the outcomes and cost-effectiveness of immigrant screening with IGRA at different incidence thresholds showed that new entrants to the UK have a high prevalence of latent infection, which varies by age, sex, and tuberculosis incidence in their country of origin (panel 2). UK national guidance for which groups to screen excludes most immigrants with latent infection, and our analysis suggests that policy could be modified in centres undertaking or considering the implementation of one-step IGRA testing to substantially reduce tuberculosis incidence while remaining cost effective.

Panel 2 **Research in context**

**Systematic review**

We searched Medline from 1960 to 2010 for studies assessing the cost-effectiveness of one-step IGRA-based screening for latent-tuberculosis infection in immigrants to high-income countries. There were no published studies that used IGRA testing to parameterise a health-economic model with the specific aim of defining the most cost-effective tuberculosis incidence threshold.

**Interpretation**

Our findings indicate that immigrants arriving in the UK, who originate from mostly countries with high burdens of tuberculosis, have a high prevalence of latent tuberculosis infection, which is strongly associated with the incidence of tuberculosis in their countries of origin. Current guidelines miss most imported cases of latent tuberculosis but screening for latent infection can be cost-effectively implemented at an incidence threshold that identifies most immigrants with latent infection, thereby preventing substantial numbers of future cases of active tuberculosis.

In our cohort, the prevalence of latent infection was moderately high at 20%. Past studies from various settings, which used tuberculin skin test to diagnose latent infection recorded 34–55% of immigrants to be skin-test-positive.^37^, ^38^, ^39^, ^40^ These high proportions are likely to show cross reactivity of past vaccination with BCG, resulting in many false-positive skin-test results.^34^ Therefore, the main implication of screening with the skin test is that an increased number of uninfected individuals will be unnecessarily treated with chemoprophylaxis. However IGRAs, which have a high specificity in BCG-vaccinated patients, result in fewer false-positives than occur with tuberculin skin tests and, therefore, might provide a reduced, but accurate, estimated prevalence of latent infection in immigrants.^34^ Data for the burden of latent infection diagnosed by IGRA in immigrants are scarce and relate generally to immigrant tuberculosis contacts or undocumented immigrants.^15^, ^37^, ^40^, ^41^, ^42^ These studies from various parts of Europe, including the UK, have suggested that 15–38% of new entrants have positive IGRA results.

Immigrants to the UK (other than from within the EU) arrive largely from countries with the highest burdens of tuberculosis.^5^ In our cohort, about 81% of all screened immigrants and just less than 87% of all cases of latent infection were from the Indian subcontinent and sub-Saharan Africa. However, by including several UK centres with different patterns of migration, we provide reliable estimates for the prevalence of latent infection in immigrants arriving from countries with a wide range of tuberculosis burden. Prevalence of latent infection was correlated independently with tuberculosis incidence in the immigrants' country of origin. No immigrants from countries with incidences less than 50 cases per 100 000 had a positive IGRA; a finding that is consistent with small-scale European studies that used only a binary classification of incidence less than or greater than 50 cases per 100 000.^37^, ^41^, ^42^

Increased age was also independently associated with an increased likelihood of a positive IGRA result.^43^ Although past work from both developed and developing countries has shown that IGRA positivity correlates with increasing age,^44^, ^45^, ^46^ with no prospective data, whether this correlation represents a truly higher prevalence of latent infection (due to more cumulative exposure in settings with high burdens of tuberculosis) or suboptimum IGRA-sensitivity in younger individuals is unclear.^47^

Evidence has shown that many areas of the UK do not follow national guidelines for screening of latent infection and have set their own criteria for screening, and, indeed, our data suggest that NICE's 2006 cutoff in 16–35-year-olds might be too high and restrictive.^13^, ^15^ If we applied national guidance (which has been in place since 2006) in our cohort to those aged 35 years and younger, only 29% of latent infections would be identified, leaving nearly three-quarters (mostly those from the Indian subcontinent) undiagnosed and at risk of developing active disease and possibly infecting others. Indeed, these immigrants from the Indian subcontinent constitute the largest proportion of foreign-born patients with tuberculosis in the UK.^8^ If the threshold incidence in the country of origin for screening 16–35-year-olds were reduced to 150/100 000, 92% of those with latent infections would be identified, leaving only a small fraction undiagnosed and at risk of developing active disease.

Our health-economic analysis indicated that for one-step IGRA screening of 16–35 year-olds, four incidence thresholds were cost effective, and all were more cost effective than the threshold that is currently recommended by national guidance. The two most cost-effective strategies were to screen at 250/100 000 and higher (with an ICER of £17 956·0 per tuberculosis case averted) and to screen at 150/100 000, which would avert an additional 29·2 cases of active disease per 10 000 immigrants (compared to screening at ≥250/100 000) at a marginally increased ICER of £20 818·8 per each additional case averted. This second strategy would encompass individuals from many Asian countries who are currently excluded, including those from the Indian subcontinent who form a large proportion of immigrants to the UK.^4^ Further reduction of the threshold to 40 cases per 100 000 or even lower (ie, screening all immigrants) would prevent further cases of active infection; however, starting screening at these reduced thresholds would incur substantially increased total costs—therefore, resource availability and the funds that policy makers are willing to spend to control the incidence of active tuberculosis would need to be reconsidered.

Past health-economic analyses compared tuberculin skin-test with chest radiography for screening new-entrants from countries with high burdens of tuberculosis (especially for active tuberculosis).^48^, ^49^ Although Schwartzman and colleagues^48^ reported that screening with chest radiographs was more cost effective than with skin tests, this conclusion might not be universally relevant because the investigators assumed that most unscreened immigrants developing active disease would need prolonged in-patient management. By contrast, Dasgupta and colleagues^49^ noted that screening and treatment of immigrants for latent infection in a subset who had undergone chest radiography and skin tests had important public health effects, but would be expensive because of poor programme efficiency (eg, the proportion of immigrants completing chemoprophylaxis). Oxlade and colleagues^50^ have compared several scenarios of immigrant screening, including chest radiography, tuberculin skin test, and IGRAs, and shown that all techniques had a modest effect on tuberculosis notifications; chest radiography alone was the most cost-effective option. However, the model was based on putative scenarios rather than on actual data, and assumed a very low prevalence of latent infection in new immigrants (0·08–2·1%) and a low rate of reactivation. Our study advances the evidence base by using unique, accurate, and empirical IGRA screening data from various centres to objectively apply parameters to a decision model for assessing the key question of yields and cost-effectiveness of immigrant screening at different levels of incidence.

Although we chose a conservative progression rate from latent tuberculosis to active tuberculosis, of 5% over 20 years, this rate remains poorly understood. Marks and colleagues^51^ calculated a progression rate of 6·7% over 40 years in tuberculosis skin-test-positive (>15 mm) refugees from southeast Asia.^47^ However, data from the UK,^52^ in a population similar to ours, suggest that over 10-years, about 13% of skin-test-positive, untreated immigrants (mostly from the Indian subcontinent) progress to active tuberculosis. These data mean that our results probably underestimate the true cost-effectiveness. Further work should ascertain whether the actual rates of disease progression in IGRA-positive immigrants after arrival and, specifically, whether this rate differs according to age and country of origin.

The success of screening will depend on implementation of robust systems, which will allow immigrants to be identified in a timely fashion; however, the overall effect of screening will be largely determined by patient and physician adherence both to having the diagnostic test, and to completing the chemoprophylactic drug regimen. A more specific blood test (ie, IGRA) might increase compliance in immigrants compared to two visits for skin tests, which are frequently false positive in this BCG-vaccinated population.^22^, ^34^

Our work had several limitations. Routine surveillance data are likely to under report the prevalence of infection, whereas any selection bias in which immigrants attended for screening could increase the prevalence of latent infection in our study. Moreover, we did not have concurrent results for tuberculin skin test against IGRA because the participating centres do not routinely do skin tests in new-entrants. One of the most substantial obstacles with test performance is the scarcity of a gold-standard test for latent tuberculosis, which makes it difficult to calculate the sensitivity of diagnostic tests for this infection. We therefore used figures from the most up-to-date meta-analysis of IGRA performance in which culture-confirmed active tuberculosis was the surrogate reference standard.^22^ Because IGRA sensitivity is likely to be lower in patients with active tuberculosis than in healthy individuals undergoing screening for latent infection, this assumption might underestimate the sensitivity of the test and therefore the cost-effectiveness estimates. By contrast, if specificity estimates are based on preselected patients with a very low probability of tuberculosis, the test specificity might be overestimated. Increased estimates would give fewer false-positive results, thereby overestimating the cost-effectiveness of screening.

In our health-economic analysis we made some assumptions about the natural history of tuberculosis (eg, onward transmission to contacts and complete clearance of infection, with no risk of reinfection after chemoprophylaxis) because this was not a formal dynamic model that would allow us to capture the intrinsic transmission dynamics of tuberculosis. Although we included secondary cases of active and latent tuberculosis, incorporation of tertiary and quaternary cases would further increase cost-effectiveness. Moreover, we did not incorporate drug-resistant strains or HIV infection. Although data from our study parameterised the model, uncertainty surrounds several variables for which we made assumptions—eg, we assumed that there were no prevalent cases of active tuberculosis in the screened cohort, but, in reality, a small proportion of individuals proved to have active disease as a result of screening. By not incorporating these factors into the decision-analysis, our analysis could underestimate the cost-effectiveness of screening. By contrast, we assumed that all patients with active disease would be diagnosed, accept, and complete, treatment, and this assumption could result in overestimation of cost-effectiveness.

Unlike NICE's cost-utility analysis in which assessments of different strategies are made using cost per quality-adjusted life-year, like other investigators, we assessed effectiveness as cost per tuberculosis case prevented,^48^, ^50^, ^53^, ^54^ because objective data on quality-adjusted life-years are still scarce for patients with active tuberculosis and for those receiving chemoprophylaxis.

As national guidelines are developed for screening of latent tuberculosis with new techniques (such as IGRA), they will need to quantitatively integrate the prevalence of latent infection in immigrant populations from different regions to formulate policy that cost-effectively improves tuberculosis control and prevention.^55^ Finally, although we assessed the cost-effectiveness of screening at different thresholds with one-step IGRA, further work should compare different screening protocols (such as skin test with IGRA *vs* skin-test alone *vs* IGRA alone) and different IGRA tests (QuantiFERON-TB Gold In-Tube *vs* T-SPOT.TB *vs* next-generation IGRA).^34^, ^56^, ^57^, ^58^, ^59^

## Web Extra Material

Supplementary webappendix

## References

[1] European Centre for Disease Prevention and Control (July, 2009). Migrant health: background note to the “ECDC report on migration and infectious diseases in the EU”. http://www.ecdc.europa.eu/en/publications/Publications/0907_TER_Migrant_health_Background_note.pdf.

[2] Health Protection Agency (October, 2010). Tuberculosis in the UK: report on tuberculosis surveillance and control in the UK 2010. http://www.hpa.org.uk/web/HPAwebFile/HPAweb_C/1287143594275.

[3] EuroTB Total TB cases and TB notification rates, 1995–2006, WHO European Region 2006. http://www.eurotb.org/slides/2008/TBCases_Rates1995-2006.pdf.

[4] Office for National Statistics Total internal migration (TIM) tables. 1991–present. http://www.statistics.gov.uk/StatBase/Product.asp?vlnk=507.

[5] Gilbert RL, Antoine D, French CE, Abubakar I, Watson JM, Jones JA (2009). The impact of immigration on tuberculosis rates in the United Kingdom compared with other European countries. Int J Tuberc Lung Dis.

[6] Maguire H, Dale JW, McHugh TD (2002). Molecular epidemiology of tuberculosis in London 1995–7 showing low rate of active transmission. Thorax.

[7] French CE, Antoine D, Gelb D, Jones JA, Gilbert RL, Watson JM (2007). Tuberculosis in non-UK-born persons, England and Wales, 2001–2003. Int J Tuberc Lung Dis.

[8] Health Protection Agency (December, 2009). Tuberculosis in the UK: annual report on tuberculosis surveillance and control in the UK 2009. http://www.hpa.org.uk/web/HPAwebFile/HPAweb_C/1259152022594.

[9] Moore-Gillon J, Davies PD, Ormerod LP (2010). Rethinking TB screening: politics, practicalities and the press. Thorax.

[10] Alvarez GG, Gushulak B, Abu Rumman K (2010). A comparative examination of tuberculosis immigration medical screening programs from selected countries with high immigration and low tuberculosis incidence rates. BMC Infect Dis.

[11] Department of Health (1992). Medical examination under the Immigration Act 1971: instructions to medical inspectors. http://www.dh.gov.uk/prod_consum_dh/groups/dh_digitalassets/@dh/@en/documents/digitalasset/dh_4111790.pdf.

[12] National Institute for Health and Clinical Excellence (March, 2006). Tuberculosis: clinical diagnosis and management of tuberculosis, and measures for its prevention and control. http://www.nice.org.uk/nicemedia/pdf/CG033niceguideline.pdf.

[13] Pareek M, Abubakar I, White PJ, Garnett GP, Lalvani A (2010). TB screening of migrants to low TB burden nations: insights from evaluation of UK practice. Eur Respir J.

[14] Mazurek GH, Jereb J, Vernon A, for the Centers for Disease Control and Prevention (CDC) (2010). Updated guidelines for using interferon gamma release assays to detect *Mycobacterium tuberculosis* infection—United States, 2010. MMWR Recomm Rep.

[15] Hardy AB, Varma R, Collyns T, Moffitt SJ, Mullarkey C, Watson JP (2010). Cost-effectiveness of the NICE guidelines for screening for latent tuberculosis infection: the QuantiFERON-TB Gold IGRA alone is more cost-effective for immigrants from high burden countries. Thorax.

[16] Lalvani A (2007). Diagnosing tuberculosis infection in the 21st century: new tools to tackle an old enemy. Chest.

[17] Lalvani A, Pathan AA, Durkan H (2001). Enhanced contact tracing and spatial tracking of *Mycobacterium tuberculosis* infection by enumeration of antigen-specific T cells. Lancet.

[18] Ewer K, Deeks J, Alvarez L (2003). Comparison of T-cell-based assay with tuberculin skin test for diagnosis of *Mycobacterium tuberculosis* infection in a school tuberculosis outbreak. Lancet.

[19] Haldar P, Thuraisingham H, Hoskyns W, Woltmann G (2009). Contact screening with single-step TIGRA testing and risk of active TB infection: the Leicester cohort analysis. Thorax.

[20] Yoshiyama T, Harada N, Higuchi K, Sekiya Y, Uchimura K (2010). Use of the QuantiFERON-TB Gold test for screening tuberculosis contacts and predicting active disease. Int J Tuberc Lung Dis.

[21] Kik SV, Franken WP, Mensen M (2010). Predictive value for progression to tuberculosis by IGRA and TST in immigrant contacts. Eur Respir J.

[22] Diel R, Loddenkemper R, Niemann S, Meywald-Walter K, Nienhaus A (2010). Negative and positive predictive value of a whole-blood IGRA for developing active TB–an update. Am J Respir Crit Care Med.

[23] Bakir M, Millington KA, Soysal A (2008). Prognostic value of a T-cell based, interferon-gamma biomarker in children with tuberculosis contact. Ann Intern Med.

[24] Leung CC, Yam WC, Yew WW (2010). T-Spot.TB outperforms tuberculin skin test in predicting tuberculosis disease. Am J Respir Crit Care Med.

[25] Doherty TM, Demissie A, Olobo J (2002). Immune responses to the *Mycobacterium tuberculosis*-specific antigen ESAT-6 signal subclinical infection among contacts of tuberculosis patients. J Clin Microbiol.

[26] Aichelburg MC, Rieger A, Breitenecker F (2009). Detection and prediction of active tuberculosis disease by a whole-blood interferon-gamma release assay in HIV-1-infected individuals. Clin Infect Dis.

[27] Lienhardt C, Fielding K, Hane AA (2010). Evaluation of the prognostic value of IFN-γ release assay and tuberculin skin test in household contacts of infectious tuberculosis cases in Senegal. PLoS One.

[28] Mahomed H, Hawkridge T, Verver S (2011). The tuberculin skin test versus QuantiFERON TB Gold in predicting tuberculosis disease in an adolescent cohort study in South Africa. PLoS One.

[29] Bakir M, Dosanjh DP, Deeks JJ (2009). Use of T cell-based diagnosis of tuberculosis infection to optimize interpretation of tuberculin skin testing for child tuberculosis contacts. Clin Infect Dis.

[30] Office for National Statistics Final mid-2009 population estimates: quinary age groups for primary care organisations in England; estimated resident population (experimental). http://www.statistics.gov.uk/statbase/product.asp?vlnk=15106.

[31] Office for National Statistics Country of birth by primary care organisation (table UV08). http://www.neighbourhood.statistics.gov.uk/dissemination/.

[32] WHO (2009). Global tuberculosis control: epidemiology, strategy, financing. http://whqlibdoc.who.int%20publications/2009/9789241563802_eng.pdf.

[33] Diel R, Loddenkemper R, Nienhaus A (2010). Evidence-based comparison of commercial interferon-gamma release assays for detecting active TB: a meta-analysis. Chest.

[34] Lalvani A, Pareek M (2009). A 100 year update on diagnosis of tuberculosis infection. Br Med Bull.

[35] Department of Health Immunisation against infectious disease. In: Salisbury D, Ramsay M, Noakes K, eds. 2006. http://www.dh.gov.uk/prod_consum_dh/groups/dh_digitalassets/@dh/@en/documents/digitalasset/dh_125349.pdf.

[36] WHO (2009). Global tuberculosis control: epidemiology, strategy, financing. http://whqlibdoc.who.int/publications/2009/9789241563802_eng.pdf.

[37] Orlando G, Merli S, Cordier L (2010). Interferon-γ releasing assay versus tuberculin skin testing for latent tuberculosis infection in targeted screening programs for high risk immigrants. Infection.

[38] Gibney KB, Mihrshahi S, Torresi J, Marshall C, Leder K, Biggs BA (2009). The profile of health problems in African immigrants attending an infectious disease unit in Melbourne, Australia. Am J Trop Med Hyg.

[39] Haley CA, Cain KP, Yu C, Garman KF, Wells CD, Laserson KF (2008). Risk-based screening for latent tuberculosis infection. South Med J.

[40] Carvalho AC, Pezzoli MC, El-Hamad I (2007). QuantiFERON-TB Gold test in the identification of latent tuberculosis infection in immigrants. J Infect.

[41] Kik SV, Franken WP, Arend SM (2009). Interferon-gamma release assays in immigrant contacts and effect of remote exposure to *Mycobacterium tuberculosis*. Int J Tuberc Lung Dis.

[42] Bodenmann P, Vaucher P, Wolff H (2009). Screening for latent tuberculosis infection among undocumented immigrants in Swiss healthcare centres; a descriptive exploratory study. BMC Infect Dis.

[43] Soysal A, Millington KA, Bakir M (2005). Effect of BCG vaccination on risk of *Mycobacterium tuberculosis* infection in children with household tuberculosis contact: a prospective community-based study. Lancet.

[44] Winje B, Oftung F, Korsvold G (2008). Screening for tuberculosis infection among newly arrived asylum seekers: Comparison of QuantiFERON TB Gold with tuberculin skin test. BMC Infect Dis.

[45] Lien LT, Hang NT, Kobayashi N (2009). Prevalence and risk factors for tuberculosis infection among hospital workers in Hanoi, Viet Nam. PLoS One.

[46] Mutsvangwa J, Millington KA, Chaka K (2010). Identifying recent *Mycobacterium tuberculosis* transmission in the setting of high HIV and TB burden. Thorax.

[47] Bamford ARJ, Crook AM, Clark JE, et al. Comparison of interferon-γ release assays and tuberculin skin test in predicting active tuberculosis (TB) in children in the UK: a paediatric TB network study. *Arch Dis Child*; **95:** 180–86. 10.1136/adc.2009.169805 19815937

[48] Schwartzman K, Menzies D (2000). Tuberculosis screening of immigrants to low-prevalence countries. A cost-effectiveness analysis. Am J Respir Crit Care Med.

[49] Dasgupta K, Schwartzman K, Marchand R (2000). Comparison of cost-effectiveness of tuberculosis screening of close contacts and foreign-born populations. Am J Respir Crit Care Med.

[50] Oxlade O, Schwartzman K, Menzies D (2007). Interferon-gamma release assays and TB screening in high-income countries: a cost-effectiveness analysis. Int J Tuberc Lung Dis.

[51] Marks GB, Bai J, Simpson SE, Sullivan EA, Stewart GJ (2000). Incidence of tuberculosis among a cohort of tuberculin-positive refugees in Australia: reappraising the estimates of risk. Am J Respir Crit Care Med.

[52] Choudry IW, Ormerod LP (2007). The outcome of a cohort of tuberculin positive, predominantly south Asian, new entrants aged 16–34 to the UK: Blackburn 1989–2001. Thorax.

[53] National Institute for Health and Clinical Excellence (March, 2011). Tuberculosis: clinical diagnosis and management of tuberculosis, and measures for its prevention and control. http://www.nice.org.uk/nicemedia/live/13422/53642/53642.pdf.

[54] Diel R, Nienhaus A, Loddenkemper R (2007). Cost-effectiveness of interferon-gamma release assay screening for latent tuberculosis infection treatment in Germany. Chest.

[55] Pooran A, Booth H, Miller RF (2010). Different screening strategies (single or dual) for the diagnosis of suspected latent tuberculosis: a cost effectiveness analysis. BMC Pulm Med.

[56] Dosanjh DP, Hinks TS, Innes JA (2008). Improved diagnostic evaluation of suspected tuberculosis. Ann Intern Med.

[57] Casey R, Blumenkrantz D, Millington K (2010). Enumeration of functional T-cell subsets by fluorescence-immunospot defines signatures of pathogen burden in tuberculosis. PLoS One.

[58] Millington KA, Fortune SM, Low J (2011). Rv3615c is a highly immunodominant RD1 (Region of Difference 1)-dependent secreted antigen specific for *Mycobacterium tuberculosis* infection. Proc Natl Acad Sci USA.

[59] Lalvani A, Millington KA (2008). T-cell interferon-γ release assays: can we do better?. Eur Respir J.

