# Supplementary material for: Screening of immigrants in the UK for imported latent tuberculosis: a multicentre cohort study and cost-effectiveness analysis
Source: Lancet Infect Dis. 2011 Jun;11(6):435–44. doi: 10.1016/S1473-3099(11)70069-X (PMC3108102; doi:10.1016/S1473-3099(11)70069-X)
Supplement: Supplementary webappendix [file mmc1.pdf]

THE LANCET Infectious Diseases

Supplementary webappendix

This webappendix formed part of the original submission and has been peer reviewed. We post it as supplied by the authors.

Supplement to: Pareek M, Watson JP, Ormerod LP, et al. Screening of immigrants in the UK for imported latent tuberculosis: a multicentre cohort study and cost-effectiveness analysis. *Lancet Infect Dis* 2011; published online April 21. DOI:10.1016/S1473-3099(11)70069-X.

Current UK immigrant screening misses the majority of imported latent tuberculosis: a multi-centre cohort study and cost-effectiveness analysis of improved screening

Manish Pareek MRCP *Clinical Research Fellow*[a,b]
John P. Watson FRCP *Consultant Physician*[c]
L Peter Ormerod FRCP *Professor*[d]
Onn Min Kon FRCP *Consultant*[e]
Gerrit Woltmann FRCP *Consultant*[f]
Peter J White PhD *Section Head*[g] *and Lecturer in Infectious Disease Epidemiology*[a]
Ibrahim Abubakar PhD *Section Head*[h] and *Senior Lecturer*[i]
Ajit Lalvani DM *Chair of Infectious Diseases and Director*[a]
[a]Tuberculosis Research Unit, National Heart and Lung Institute, Imperial College London, UK
[b]Department of Infectious Disease Epidemiology, Imperial College London, UK
[c]Department of Respiratory Medicine, Leeds Teaching Hospitals NHS Trust, UK
[d]Chest Clinic, Royal Blackburn Hospital, Blackburn, UK
[e]Tuberculosis Service, Chest and Allergy Clinic, St. Mary's Hospital, Imperial College Healthcare NHS Trust, UK
[f]Department of Respiratory Medicine, Glenfield Hospital, University Hospitals Leicester NHS Trust, UK
[g]Modelling and Economics Unit, Centre for Infections, Health Protection Agency, London, UK
[h]Tuberculosis Section, Centre for Infections, Health Protection Agency, London, UK
[i]School of Medicine, Health Policy and Practice, University of East Anglia, UK

Corresponding author:
Professor Ajit Lalvani DM FRCP
Director, Tuberculosis Research Unit
National Heart and Lung Institute
Imperial College London
Norfolk Place, London, UK W2 1PG
Email: a.lalvani@imperial.ac.uk Tel: +44 (0) 207 594 0883

Description of decision model

The decision model considers a hypothetical cohort of 10,000 recent immigrants, aged ≤35, arriving in the United Kingdom. Eligibility for LTBI screening is predicated on the migrant's age (whether <16 or 16-35 years) and tuberculosis (TB) incidence in their country of origin (using increments of 50/100,000) as shown in online supplementary table 1.

Depending on the incidence threshold at which screening is instigated, a proportion of these migrants are eligible to be screened and undergo interferon gamma release assay (IGRA) testing which will yield either a positive or negative result. In our multi-centre cohort, which we use to parameterise our model, the proportion of indeterminate results was low (0.16%). Therefore, we assumed that the indeterminate rate was negligible in this hypothetical model cohort. A proportion of the immigrants who are not eligible to be tested at a specific threshold will also have LTBI and remain at risk of progressing to active TB; those immigrants who are ineligible, and uninfected, have no further sequelae in the model.

Individuals who are IGRA positive (which can be either true or false positive) will go on to be assessed for active TB with chest radiography. As a simplification it is assumed that there are no prevalent cases of active TB identified in the immigrant cohort at the time of screening. In reality, however, a very small proportion of migrants will have active TB (0.3% in our cohort). Nonetheless, this is extremely rare and in most cases, the chest radiograph/assessment for active TB will be normal and the immigrant will be offered chemoprophylaxis with 3 months of rifampicin and isoniazid.

A proportion of the true and false positive individuals commence therapy. Amongst these individuals who do commence therapy, a proportion will develop drug-induced liver injury even though this is rare (0.2%[1]) in the age-group we considered (immigrants ≤35 years). In those who develop drug induced liver injury (DILI), extra costs in the form of additional clinic visits, inpatient admission and blood tests are required. Eventually, chemoprophylaxis will need to be stopped in a small proportion as the drug induced liver injury will not improve. It is assumed that these individuals will have only completed four weeks of therapy and so the efficacy of the chemoprophylaxis is negligible. Therefore these individuals remain latently infected and thus at future risk of progressing to active TB disease. However, in the vast majority, the drug induced liver injury will usually improve. In those individuals where the liver function tests return to normal, treatment will be (re)continued and defined proportions will complete and not complete therapy.

In those individuals who do not develop DILI, uncomplicated treatment will ensue with fixed proportions completing and not completing therapy.

Complete treatment will have a 65% efficacy in preventing progression from LTBI to active TB.[2] This means that there will be some patients who complete therapy but in whom the drug regimen has been ineffectual. As an added layer of complexity it is assumed that the proportion of immigrants in whom hepatotoxic effects have improved but do not complete therapy will have completed 50% of the drug regimen (ie. 6 weeks) and, based on data suggesting equivalence of 3 months of rifampicin and isoniazid and 6 months isoniazid, we assume, in keeping with previous authors[3 4], that this reduces the risk of reactivation by 21%.[2] It is important to note that therapy is only efficacious in truly infected individuals and is of no benefit (in terms of preventing reactivation to active TB) for those immigrants who test false-positive with the IGRA.

In those individuals, who are true positive, who have completed chemoprophylactic therapy (either fully or partially) which has been successful we assume that infection with *Mycobacterium tuberculosis* is cleared ("Clearance of M. tuberculosis infection") with no further sequelae for these individuals. However, if therapy has not been successful ("Non-clearance of M. tuberculosis infection") it is assumed that the individuals have not cleared *M. tuberculosis* infection and so remain latently infected. Over the time horizon of the model, these individuals can either "remain in LTBI state" or "reactivate to active TB disease". False-positive individuals, whether or not they completed the therapeutic course, are not actually infected and so there are no further sequelae.

Immigrants with negative IGRA results are assumed to be uninfected and will be discharged from the screening clinic. For those individuals who are truly uninfected no further costs or effects are incurred following screening. In contrast, individuals who are false-negative actually have LTBI and are at risk of progressing to active TB disease (with its attendant costs) over the 20-year horizon of the model. In fact, it is important to note that a number of different groups within the immigrant cohort will have LTBI and remain at risk of progressing to active TB disease over the time horizon of the model including:

1. Immigrants with LTBI who are not eligible to be screened as they originate from a country with a TB incidence which does not meet the screening criteria (eg. an individual arriving

from India (TB incidence 170/100000) but screening is limited to individuals arriving from countries with a TB incidence ≥200/100,000.
2. Immigrants with LTBI who screen positive but decline to commence chemoprophylaxis.
3. Immigrants with LTBI who screen positive, accept chemoprophylaxis, do not develop hepatotoxicity, complete therapy but it is not effective.
4. Immigrants with LTBI who screen positive, accept chemoprophylaxis, do not develop hepatotoxicity do not complete therapy which is not effective.
5. Immigrants with LTBI who screen positive, accept chemoprophylaxis, develop hepatotoxicity which resolves, complete therapy but it is not effective.
6. Immigrants with LTBI who screen positive, accept chemoprophylaxis, develop hepatotoxicity which resolves, do not complete therapy but it is not effective.
7. Individuals with LTBI who screen positive, accept chemoprophylaxis, develop hepatotoxicity which does not resolve resulting in them stopping therapy early. This renders them still latently infected.
8. Immigrants with LTBI but who actually test false-negative with the IGRA.

These individuals remain at risk of progressing from the latent state to active TB disease at a fixed rate. In the absence of reliable data about the proportion of migrants with HIV infection it is assumed that none of the immigrants have HIV.

If an individual with LTBI breaks down to active TB disease all strains are assumed to be fully drug sensitive. Individuals with active TB are modelled to have a fixed number of contacts which will result in a fixed number of secondary active TB cases and LTBI. Depending on the severity of disease, a proportion of individuals will need to be hospitalised whilst the remainder will be managed as outpatients. It is assumed that all subjects accept treatment and that treatment for all cases of active TB follows national guidelines with compliance, and cure, fixed at 100%. Once an individual has been treated for active TB they cannot be re-infected during the course of the 20 year model. In view of the low mortality rate from TB in the UK it is assumed that there is no TB/background mortality during the 20-year horizon of the model.

Input parameters and probabilities

Input data were obtained from the present multi-centre study whilst probabilities for transitioning between states were obtained from previous literature(see online supplementary table 2).

Key to the cost-effectiveness analysis were the parameters used to describe the performance of the IGRA (QuantiFERON-Gold In-tube) in diagnosing LTBI. These were obtained from the most recent meta-analysis on IGRA performance which concluded the QuantiFERON Gold In-tube has a specificity of 99% and sensitivity, in developed countries, of 84%.[5] The high specificity means that the proportion of false-positive results is relatively small, given the relatively high prevalence of LTBI in the cohort. [4 6] In contrast, the sensitivity of the IGRA impacts on the proportion of false-negative results.

When calculating the true prevalence of LTBI in the tested cohort, it is important to take into account test performance. If, for example, 20% of individuals are IGRA positive it would be incorrect/inaccurate to simply assume that these 20% represent all truly infected individuals. The reason for this is that, depending on test sensitivity and specificity, a proportion of positives will be falsely-positive whilst some negatives will be falsely-negative. We therefore calculated the true prevalence of LTBI in the cohort by using the following formula:

*Probability of a positive result = (Test sensitivity*Prevalence of LTBI) + ((1-Test specificity)*(1-Prevalence))*

Rearranging for Prevalence of LTBI, the formula becomes:

Prevalence of LTBI = *Probability of a positive result – (1-Test specificity)/((Test sensitivity)-(1-Test specificity))*

This is important because, returning to our example of 20% of individuals being IGRA positive when test performance suggests a sensitivity of 84% and specificity of 99%, the true prevalence is actually 22.9%.

Another important, but poorly understood, parameter was the rate at which immigrants with LTBI reactivated and progressed to active TB disease. Although immigrants should, in theory, have a

lower rate of progression than recent contacts of smear-positive tuberculosis, it could be argued that those individuals arriving from high TB burden countries are, in fact, akin to recent contacts as they will have been recently and repeatedly exposed to individuals with infectious tuberculosis. This makes it difficult to parameterise the progression rate with full certainty. For example whilst Marks et al calculated 6.7% progression over a 40 year period in TST positive (>15mm) Southeast Asian refugees[7], data from the UK, in a predominantly Southeast Asian population suggests that over a 10 year period approximately 13% of TST positive, untreated, immigrants (primarily from the Indian Subcontinent) will go on to develop active TB.[8] Horsburgh estimated that in 16-35 year olds with a >15mm TST (not recently converted) the annual risk of reactivation was 0.19%.[9] If the skin test was >15mm and there was recent conversion then the annual risk of reactivation would 0.56%.[9] In view of the large difference in published data we assumed that 5% of the cohort with LTBI, in the absence of chemoprophylaxis, would progress to active TB over the 20 year time horizon; a suitably wide range was explored in the sensitivity analysis.

Costs
Component costs considered were primarily direct costs obtained from economic evaluations conducted for the UK NICE TB guidelines[1], and its forthcoming update, uplifted to 2010 prices(see table 2 for costs) using the Consumer Prices Index. In the present analysis, indirect costs such as transportation and loss of earnings by patients were not considered. Both costs and non-monetary health effects were discounted at an annual rate of 3.5%, which reflects UK Treasury and NICE recommendations.[10 11]

Effects
The main effects considered in the model were the number of cases of active tuberculosis that would be predicted to occur over the 20-year time horizon and the number needed to treat (in other words the number of individuals that need to be treated for LTBI) to prevent one case of active TB.

Cost-effectiveness
As recommended by the Panel of Cost-effectiveness in Health and Medicine, the comparative performance of the different screening protocols was measured using the Incremental Cost-effectiveness ratio (ICER – see equation 1) which quantifies the trade-offs between switching from one competing, mutually-exclusive, intervention to another.[12] The higher the ICER, the less cost-effective the intervention is.

$$ICER = \frac{Cost_{screeningprotocolA} - Cost_{screeningprotocolB}}{Effectiveness_{screeningprotocolA} - Effectiveness_{screeningprotocolB}}$$

Sensitivity analysis
Parameter uncertainty can potentially affect the results of the cost-effectiveness analysis. A simple one-way sensitivity analysis was therefore undertaken to explore the impact that changes in all key parameters and costs had on the number of cases of active TB occurring over 20 years, the costs and the associated ICERs.

Supplementary table 1. Screening thresholds considered in the cost-effectiveness analysis

Screening threshold for immigrants (number of cases of TB/100,000)[1]	
**Under 16**	**16-35 years**
None	None
40	500
40	450
40	400
40	350
40	300
40	250
40	500+SSA[2]
40	200
40	150
40	100
40	40
All	All

[1]Refers to TB incidence in the country of origin
[2]Sub-Saharan Africa

Supplementary table 2. Parameters used as input values in the decision model

Probabilities	Base case (range)[1]	References
IGRA positivity/prevalence of LTBI in under 35 year old cohort	Varies (table 2 in main text)	Current study
Proportion undergoing screening	Varies (table 3 in main text)	
Specificity of QuantiFERON	0.99 (0.9-1.0)	5 6
Sensitivity of QuantiFERON	0.84 (0.78-0.90)	5
Proportion of new entrants with pre-existing active TB (ie. prevalent cases)	0.0	Assumed
Proportion of IGRA positive new entrants accepting chemoprophylaxis	0.95 (0.3-1.0)	13
Proportion of IGRA positive new entrants completing chemoprophylaxis	0.85 (0.3-1.0)	14
Duration of chemoprophylaxis with rifampicin and isoniazid (course completed)	3 months	11
Duration of chemoprophylaxis with rifampicin and isoniazid (course partially completed)	1.5 months	Assumed
Efficacy of 3 months of Rifampicin and Isoniazid	0.65 (0.5-0.8)	2 15
Efficacy of 1.5 months of Rifampicin and isoniazid (partial chemoprophylaxis)	0.21 (0.1-0.3) [2]	2
Proportion of IGRA positive individuals progressing to active TB (post-exposure TB) over 20 years	0.05 (0.025-0.10)	9
Proportion of those who are cured of LTBI who can be reinfected	0	Assumed
Proportion of those who are cured of LTBI who can reactivate to active TB	0	Assumed
Proportion of those who are not cured of LTBI who can reactivate to active TB (annually)	0.0025	9
Proportion of individuals on chemoprophylaxis who develop hepatoxicity	0.002	1
Proportion of individuals on chemoprophylaxis who develop hepatoxicity which resolves	0.9	Assumed
Proportion of individuals on chemoprophylaxis requiring inpatient hospital stay	0.2	4 16
Proportion of individuals with active TB accepting treatment	1.0	Assumed
Proportion of individuals with active TB completing therapy	1.0	Assumed
Proportion of individuals with active TB cured	1.0	Assumed
Discount rate	0.035	10

[1]Refers to the range explored in the univariate sensitivity analysis
[2]Data from IUAT study was the basis of an assumed estimate[2]
3Estimates for the proportion of IGRA positive individuals progressing to active TB drawn from Horsburgh et al which is based on TST data[9]

Supplementary table 3. Costs (2010 pounds sterling) associated with diagnosis and treatment of active TB

Active TB cases	Base case (range)[1]	Reference(s)
**Contact tracing**<br>Contact tracing per contact (£)	482 (241-723)	[14]
Mean number of contacts examined per primary case	6.5 (3.25-10)	[14]
Mean number of secondary active TB cases per index case	0.2 (0.1-0.3)	[14]
Mean number of latent infections per primary case of active TB disease	0.18 (0.09-0.27)	[17]
**Inpatient care**<br>Cost of inpatient episode for acute TB (£ per spell)	4012.97 (2006.5-6019.46)	[14 18 19]
Proportion of patients with acute TB who are admitted	0.53 (0.265-0.795)	[14]
Cost of inpatient care (£ per active case)	2126.87	Calculated
**Cost of tests**<br>Costs of culture test (£ per test)	10	[14]
Costs of chest X-ray (£ per X-ray)	28	[13]
Costs of liver functions tests	1	[13]
Culture tests per case treated	4	[13 14]
Chest X-ray per case treated	2	[13 14]
Liver functions tests per case treated	4	[13 14]
Total cost of tests (£ per TB case treated)	100[3]	Calculated
**Cost of chemotherapy**<br>Rifampicin (£ per month)	10.76	[13 14]
Isoniazid (£ per month)	17.87	[13 14]
Pyrazinamide (£ per month)	6.88	[13 14]
Ethambutol (£ per month))	18.48	[13 14]
Duration of rifampicin (months)	6	[13 14]
Duration of isoniazid (months)	6	[13 14]
**Outpatient care**<br>Cost of outpatient consultation (first visit)	257	[13 14]
Cost of outpatient consultation (follow-up visit)	130	[13 14]
Cost of TB Nurse home visit	22	[13 14]
Number of outpatient clinic visits per case treated	4	[13 14]
Visits from TB Nurse per case treated	6	[13 14]
Total costs of non-drug outpatient care (£ per case treated)	779[3]	Calculated

[1]Refers to the range explored in the univariate sensitivity analysis

[2]In the sensitivity analysis the costs of outpatient care for an active TB case and the tests required were considered together (total = 879, range explored 439.5-1318.5).

Supplementary table 4. Costs (2010 pounds sterling) associated with diagnosis and treatment of latent TB infection

**Active TB cases**	**Base case (range)[1]**	**Reference(s)**
**Cost of screening**<br>QuantiFERON (test kit, consumables and phlebotomy)	45 (22.5-90)	[13 14]
**Cost of evaluating positive QuantiFERON**<br>Cost of outpatient consultation: first visit (£ per visit)<br>CXR<br>LFT<br>Number of outpatient consultation<br>Number of CXR<br>Number of LFT	 257<br>28<br>1<br>1<br>1<br>1	[13 14]
Total cost of evaluating positive QuantiFERON	286 (143-429)	Calculated
**Cost of follow-up and chemoprophylactic therapy for positives undergoing treatment**<br>Follow up via TB nurses	22	[13 14]
Rifampicin (£ per month)	10.76	[13 14]
Isoniazid (£ per month)	17.87	[13 14]
Number of TB Nurses appointments	2	[13 14]
Duration of rifampicin (months)	3	[13 14]
Duration of isoniazid (months)	3	[13 14]
Total cost of chemoprophylaxis (£ per course) plus TB nurse follow-up	130 (65-195)	Calculated
**Cost of managing a case of chemoprophylaxis induced liver injury**<br>Cost of additional clinic visits and blood tests	362	[20]
Cost of inpatient stay	1000	[20]

[1]Refers to the range explored in the univariate sensitivity analysis

Supplementary table 5. Univariate sensitivity analysis of costs (2010 GB pounds) used as input variables in the decision model. (The figures presented are the incremental cost-effectiveness ratios (ICERs); moving from lowest to highest ICER indicates decreasing cost-effectiveness. ED = Extended dominance - this the situation where the incremental cost-effectiveness ratio (ICER) for a particular screening threshold is higher than for the next most effective strategy (screening threshold) and so the higher ICER is removed from the cost-effectiveness analysis.

Parameter	Screening thresholds for <16 and 16-35 (UK pound sterling per case averted)											
	<16 All 16-35 All	<16 40 16-35 40	<16 40 16-35 100	<16 40 16-35 150	<16 40 16-35 200	<16 40 16-35 250	<16 40 16-35 300	<16 40 16-35 350	<16 40 16-35 400	<16 40 16-35 450	<16 40 16-35 500	<16 40 16-35 500+SSA
**Cost of initial screening**												
22.50	44,942.7	20,771.3	ED	15,907.0	ED	14,357.4	ED	ED	SD	ED	ED	ED
90	215,929.6	46,666.7	ED	30,642.5	ED	25,153.2	ED	ED	SD	ED	ED	ED
**Cost of evaluating of IGRA positive new-entrants**												
143	103,960.2	22,806.1	ED	14,629.5	ED	11,850.9	ED	ED	SD	ED	ED	ED
429	99,916.4	36,000.0	ED	27,008.2	ED	24,061.1	ED	ED	SD	ED	ED	ED
**Cost of treating new-entrants**												
65	102,754.9	26,774.5	ED	18,353.2	ED	15,524.0	ED	ED	SD	ED	ED	ED
195	101,121.7	32,031.7	ED	23,284.5	ED	20,387.9	ED	ED	SD	ED	ED	ED
**Cost of active TB OP follow-up**												
439.5	102,377.8	29,842.6	ED	21,258.3	ED	18,395.5	ED	ED	SD	ED	ED	ED
1318.5	101,498.8	28,963.6	ED	20,379.3	ED	17,516.5	ED	ED	SD	ED	ED	ED
**Cost of active TB OP drugs**												
111	102,049.3	29,514.1	ED	20,929.8	ED	18,067.0	ED	ED	SD	ED	ED	ED
333	101,827.3	29,292.1	ED	20,707.8	ED	17,845.0	ED	ED	SD	ED	ED	ED
**Cost of active TB inpatient treatment**												
2006.5	103,001.7	30,466.5	ED	21,882.3	ED	19,019.4	ED	ED	SD	ED	ED	ED
6019.46	100,874.9	28,339.6	ED	19,755.4	ED	16,892.5	ED	ED	SD	ED	ED	ED
**Cost of contact tracing**												
241	103,504.8	30,969.6	ED	22,385.3	ED	19,522.5	ED	ED	SD	ED	ED	ED
723	100,371.8	27,836.6	ED	19,252.3	ED	16,389.5	ED	ED	SD	ED	ED	ED
**Cost of Hepatotoxicity with chemoprophylaxis**												
181	101,948.5	29,369.7	ED	20,787.5	ED	17,925.1	ED	ED	SD	ED	ED	ED

1862	101,903.4	29,517.0	ED	20,925.8	ED	18,061.4	ED	ED	SD	ED	ED	ED

Figures

Supplementary figure 1a. Decision tree used for the health economic analysis (Individuals fully cured of LTBI are assumed to remain free of further infection for the 20-year time horizon of the model. *For clarity all "reactivate to active TB subtrees" are shown in figure 1b below). Please note that as data is available on all migrants we are able to compute, at each incidence threshold, the number of migrants screened/not screened and subsequently the proportions that are IGRA positive and IGRA negative).

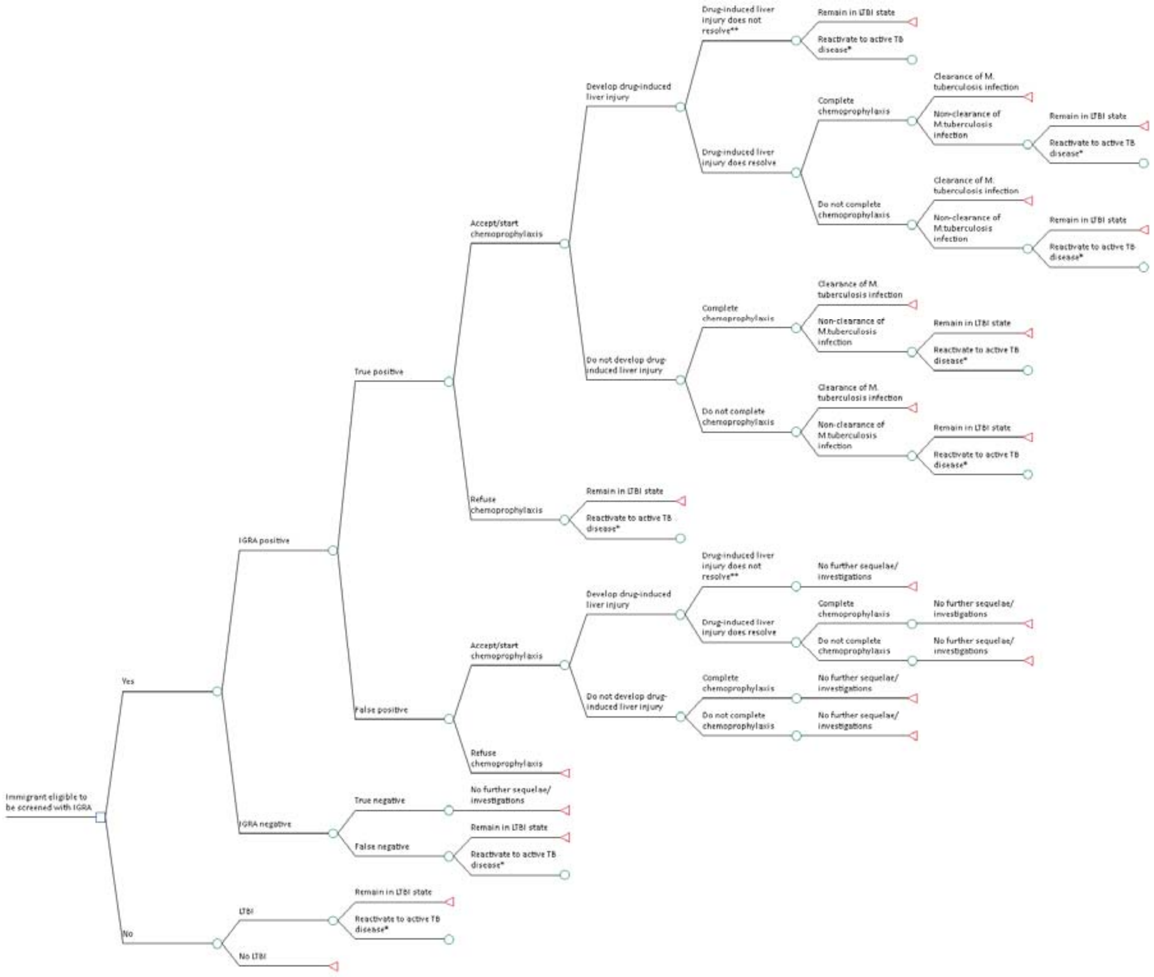

Immigrant eligible to be screened with IGRA
Yes
No
IGRA positive
IGRA negative
True positive
False positive
True negative
False negative
LTBI
No LTBI
Accept/start chemoprophylaxis
Refuse chemoprophylaxis
Develop drug-induced liver injury
Do not develop drug-induced liver injury
Drug-induced liver injury does not resolve**
Drug-induced liver injury does resolve
Complete chemoprophylaxis
Do not complete chemoprophylaxis
Clearance of M. tuberculosis infection
Non-clearance of M.tuberculosis infection
Remain in LTBI state
Reactivate to active TB disease*
No further sequelae/ investigations

Footnotes:

1. Individuals are only eligible for screening if they originate from a country which has a TB incidence equal or greater to the screening threshold selected.
2. In the model all individuals who are fully cured of LTBI are assumed to have cleared infection with *Mycobacterium tuberculosis (a terminal node)*
3. Not cured LTBI individuals remain at risk of progressing to active TB in the future
4. Individuals who develop hepatotoxicity after starting chemoprophylaxis but which subsequently does not resolve are assumed to stop the drug. In the model it is assumed that they complete only 4 weeks of treatment and this has neglible efficacy thereby leaving them latently infected and thus at future risk of reactivating to active TB
5. Individuals who develop hepatotoxicity after starting chemoprophylaxis but which subsequently resolves are assumed to continue the drug. In the model it is assumed that they can either complete or not complete treatment with their attendant outcomes

Supplementary figure 1b. Decision subtree used to describe the events that occur if an individual reactivates to active TB (although we have shown a branch/node for not accepting treatment for active TB, in the model we assume that all individuals do accept treatment for active TB)

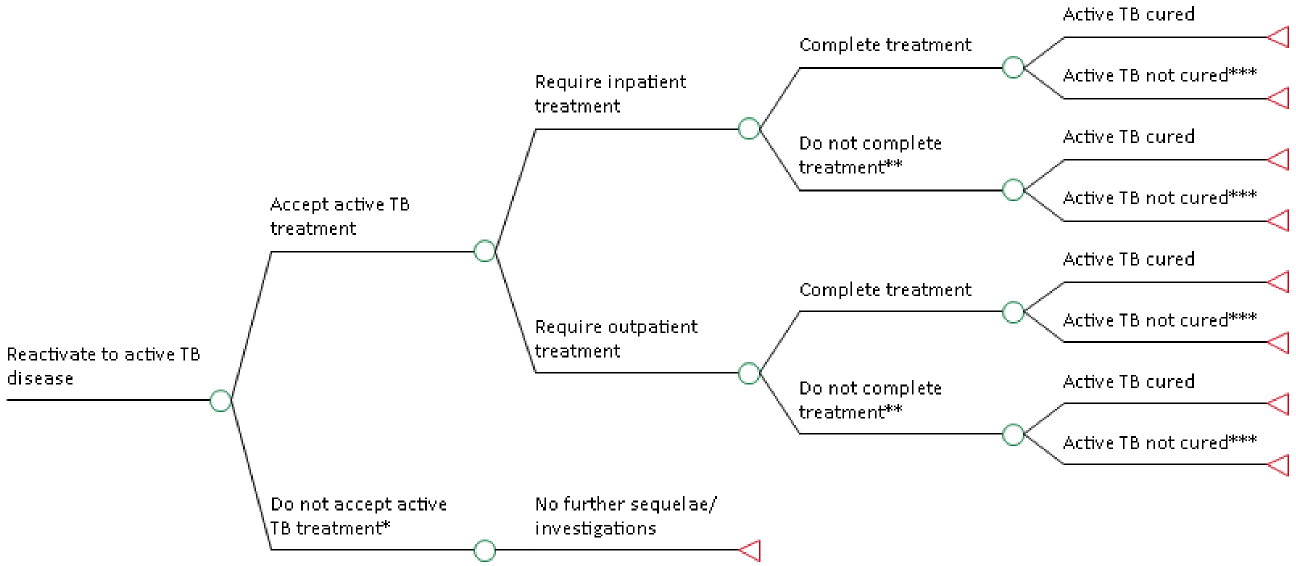

Footnotes:

Individuals who do not accept treatment(*) are represented in the model for completeness but, in reality, it is assumed that no individuals refuse treatment for active TB (ie. this is a terminal node)

Individuals who do not complete treatment(**) are represented in the model for completeness but, in reality, it is assumed that all patients complete treatment

Individuals who are active TB not cured(***) are represented in the model for completeness but, in reality, it is assumed that as they have all completed treatment none of them move into the active TB not cured group

References

1. Kunst H, Khan KS. Age-related risk of hepatotoxicity in the treatment of latent tuberculosis infection: a systematic review. *Int J Tuberc Lung Dis* 2010;14:1374-1381.
2. World Health Organisation. Efficacy of various durations of isoniazid preventive therapy for tuberculosis: five years of follow-up in the IUAT trial. International Union Against Tuberculosis Committee on Prophylaxis. *Bull World Health Organ* 1982;60:555-564.
3. de Perio MA, Tsevat J, Roselle GA, Kralovic SM, Eckman MH. Cost-effectiveness of interferon gamma release assays vs tuberculin skin tests in health care workers. *Archives of internal medicine* 2009;169(2):179 - 187.
4. Pooran A, Booth H, Miller R, Scott G, Badri M, Huggett J, et al. Different screening strategies (single or dual) for the diagnosis of suspected latent tuberculosis: a cost effectiveness analysis. *BMC Pulmonary Medicine* 2010;10(1):7.
5. Diel R, Loddenkemper R, Nienhaus A. Evidence-Based Comparison of Commercial Interferon-Gamma Release Assays for Detecting Active TB. *Chest* 2010;137(4):952-968.
6. Diel R, Nienhaus A, Loddenkemper R. Cost-effectiveness of Interferon-γ Release Assay Screening for Latent Tuberculosis Infection Treatment in Germany. *Chest* 2007;131(5):1424-1434.
7. Marks GB, Bai JUN, Simpson SE, Sullivan EA, Stewart GJ. Incidence of Tuberculosis among a Cohort of Tuberculin-Positive Refugees in Australia . Reappraising the Estimates of Risk. *Am. J. Respir. Crit. Care Med.* 2000;162(5):1851-1854.
8. Choudry IW, Ormerod LP. The outcome of a cohort of tuberculin positive, predominantly South Asian, new entrants aged 16-34 to the UK: Blackburn 1989-2001. *Thorax* 2007;62(S49):A22.
9. Horsburgh CR, Jr. Priorities for the Treatment of Latent Tuberculosis Infection in the United States. *N Engl J Med* 2004;350(20):2060-2067.
10. HM Treasury. *The Green Book: Appraisal and Evaluation in Central Government*. London: HM Treasury, 2003.
11. National Collaborating Centre for Chronic Conditions. *Tuberculosis: clinical diagnosis and management of tuberculosis, and measures for its prevention and control.* London: Royal College of Physicians, 2006.
12. Weinstein M, Siegel J, Gold M et al. Recommendations of the Panel on Cost-effectiveness in Health and Medicine. *JAMA* 1996;276:1253-1258.
13. National Collaborating Centre for Chronic Conditions. *TB Partial Update: Cost-effectiveness analysis of interferon gamma release assay (IGRA) testing for latent tuberculosis*. London: NICE, 2010.
14. National Collaborating Centre for Chronic Conditions. *Tuberculosis: appendices*. London: Royal College of Physicians, 2006.
15. Smieja MJ, Marchetti CA, Cook DJ, Smaill FM. Isoniazid for preventing tuberculosis in non-HIV infected persons. *Cochrane database of systematic reviews (Online)* 2000(2):CD001363.
16. Schwartzman K, Menzies D. Tuberculosis Screening of Immigrants to Low-Prevalence Countries . A Cost-effectiveness Analysis. *Am. J. Respir. Crit. Care Med.* 2000;161(3):780-789.
17. Underwood BR, White VL, Baker T, Law M, Moore-Gillon JC. Contact tracing and population screening for tuberculosis--who should be assessed? *J Public Health Med* 2003;25(1):59-61.
18. Office for National Statistics. Focus on Consumer Price Indices: Office for National Statistics, 2001.
19. Department of Health. *NHS Reference Costs 2005-2006*. London: Department of Health, 2006.
20. Department of Health. *Confirmation of Payment by Results (PbR) arrangements for 2010-11*. London: Crown, 2010.
